# Thrombose und COVID-19

**DOI:** 10.1007/s12326-021-00438-6

**Published:** 2021-04-15

**Authors:** Stanislava Tzaneva

**Affiliations:** Universitätsklinik für Dermatologie, Medizinische Universität Wien, Währinger Gürtel 18–20, 1090 Wien, Österreich

**Keywords:** SARS-CoV‑2, Pandemie, Venöse Thromboembolie, Koagulopathie, Inflammation, SARS-CoV‑2, Pandemic, Venous thromboembolism, Coagulopathy, Inflammation

## Abstract

Die Prävalenz der venösen thromboembolischen (VTE) Ereignisse ist bei Coronavirus diesease 2019 (COVID-19) -Patienten hoch, insbesondere bei schwer Erkrankten. Patienten mit schwerer COVID-19 und VTE haben eine signifikant höhere Mortalität im Vergleich zu Patienten ohne VTE. Die Manifestation einer schweren Infektion mit Severe acute respiratory syndrome coronavirus-2 (SARS-CoV‑2) entspricht einem systemischen proinflammatorischen und prokoagulatorischen Phänotyp, der mit vaskulären Thrombosen nicht nur in den Venen, sondern auch in den Arterien, Kapillaren sowie mit einer Inflammation der Gefäße assoziiert ist. Ein erhöhter D‑Dimer-Spiegel kann als Indikator für VTE bei Patienten mit COVID-19 verwendet werden. Die meisten medizinischen Gesellschaften empfehlen eine VTE-Prophylaxe vorzugsweise mit niedermolekularen Heparinen (LMWH) bei allen stationären Patienten. Weitere Daten von randomisierten kontrollierten Studien (RCTs) über die optimale Antikoagulation und antithrombotische Therapie werden in der nahen Zukunft erwartet.

## Abstract (en)

The prevalence of venous thromboembolism (VTE) events in Coronavirus diesease 2019 (COVID-19) patients is high, especially among those with severe disease. Patients with severe COVID-19 and VTE have significantly greater mortality compared with patients without VTE. The manifestation of a severe Severe acute respiratory syndrome coronavirus-2 (SARS-CoV‑2) infection is the systemic proinflammatory and procoagulant phenotype, associated with vascular thrombosis not only in veins, but also in arteries, capillaries and blood vessel inflammation. An increased D‑dimer level can be used as an indicator for VTE in patients with COVID-19. Medical societies have issued guidelines and recommend applying thromboprophylaxis, preferably with low molecular weight heparin (LMWH) in all patients during the hospital stay. Further evidence from ongoing randomized controlled trials (RCTs) of optimal anticoagulant and antithrombotic regimes is expected in the near future.

Wir befinden uns mitten in einer Pandemie und werden täglich mit verschiedenen Botschaften konfrontiert wie: „Halten Sie Abstand“, „Waschen Sie Ihre Hände“, „Tragen Sie eine Filtering Face Piece 2(FFP2)-Maske“. Am Anfang der Pandemie im März/April 2020 lautete die wichtigste Botschaft: „Bleiben Sie zu Hause, so können Sie Leben retten (bleiben Sie gesund).“ Viele sind dieser Botschaft gefolgt, auch viele unserer Patienten, was dazu geführt hat, dass es bei einigen zur Verschlechterung oder Progredienz von bestehenden chronischen Erkrankungen gekommen ist.

Die Pandemie hat weitreichende Folgen nicht nur in unserem Privatleben, sondern auch im Gesundheitssystem. Es ist zu Absagen vieler elektiver Eingriffe, zu Behandlungsverzögerungen oder zur suboptimalen Therapie vieler Erkrankungen gekommen. Darüber hinaus kommt es durch verstärkten Einsatz von Homeoffice und durch die mangelnde Bewegung durch die Lockdowns zur Verschlechterung von vielen chronischen Erkrankungen und zur Risikosteigerung für andere Erkrankungen.

Immer öfter sind wir mit neuen Entitäten wie der inflammatorischen Thrombose konfrontiert

Nicht zu unterschätzen sind auch die psychologischen Folgen der Pandemie mit all ihren Konsequenzen. Immer öfter sind wir mit neuen Entitäten wie der inflammatorischen Thrombose konfrontiert. Zuletzt gab es rezente Berichte über Blutungen, Thrombosen, Thrombozytopenie und einige Todesfälle nach Impfung mit COVID-19 Vakzinen, was zu Unsicherheiten bezüglich der Sicherheit den genetischen Vakzinen geführt hat. Fast alle dieser Fälle betrafen Frauen jünger als 55 Jahre und traten innerhalb der ersten sieben bis vierzehn Tage nach der Impfung auf. Die meisten dieser Fälle ereigneten sich nach Impfung mit der COVID-19 Vaccine Oxford/AstraZeneca. Dies führte zur vorübergehenden Aussetzung der Oxford/AstraZeneca Impfung in mehreren europäischen Ländern. Daraufhin haben die Medicine and Healthcare Regulatory Authority, United Kingdom (MHRA, UK) und European Medicines Agency (EMA) eine wissenschaftliche Untersuchung der Pharmakovigilanz Daten und der verfügbaren Evidenz gestartet um die potentielle Assoziation der Vakzine mit den thrombotischen Ereignissen zu beurteilen [1]. Als Ergebnis teilte EMA mit, dass das Auftreten von sehr seltenen Fällen an Thrombosen assoziiert mit einer Thrombozytopenie, mit oder ohne Blutung, einschließlich seltenen Fällen von Sinus Venen Thrombose mit der Impfung im Zusammenhang stehen könnte [2]. Gleichzeitig bestätigte EMA, dass die Vorteile der AstraZeneca Vaccine immer noch die Risiken überwiegen. Ein kontinuierliches Monitoring der Situation und weitere Überprüfung des Thrombose Risikos von anderen COVID-19 Vaccinen wird fortgeführt. Zusätzlich wurden Symptome zusammengefasst, die zu einer ärztlichen Vorstellung der Geimpften führen sollen, falls sie nach einer Impfung auftreten [2].

Mittlerweile ist bekannt, dass COVID-19 eine Multisystemerkrankung ist, die besonders bei schweren Verläufen mit einer Dysregulation des Immunsystems und Hyperkoagulation einhergeht. Die Immunothrombose, die in diesen Fällen auftritt stellt eine Interaktion zwischen dem angeborenen Immunsystem und dem Koagulationssystem dar [3].

Es gibt mittlerweile mehrere Review-Artikel und Metaanylysen, die zeigen, dass die venöse Thromboembolie eine häufige Komplikation bei hospitalisierten COVID-19-Patienten ist. Die thrombotischen Manifestationen bei COVID-19 äußern sich nicht nur als klassische tiefe Venenthrombose oder pulmonale Embolie, sondern auch als arterielle Thrombosen wie Myokardinfarkt und Insult, aber vor allem als Mikrothrombosen in vielen Geweben und Organen [4–9].

Die Gesamtinzidenz der VTE bei hospitalisierten Patienten mit COVID-19 ist, aufgrund der Heterogenität der publizierten Daten, nicht genau bekannt. Laut einer rezenten Metaanalyse beläuft sich die Inzidenz der VTE auf 38 % bei Intensivpatienten und auf 17 % bei Patienten auf Nicht-Intensivstationen. Die Inzidenz der tiefen Beinvenenthrombose (TBVT) wurde mit 22 % bei Intensivpatienten und mit 13 % bei Nicht-Intensivpatienten berichtet. Die respektiven Zahlen für die Inzidenz der Pulmonalembolie waren 22 % vs. 13 % [6].

Ein anderes rezentes systematisches Review hat eine sehr hohe Prävalenz der VTE bei Intensivpatienten von fast 50 % gezeigt, wenn sie auf eine VTE systematisch untersucht wurden [10]. Auf der anderen Seite gibt es keine Daten über die Inzidenz der VTE bei ambulanten Patienten mit COVID-19. Eine interessante retrospektive Studie lieferte Hinweise, dass auch recht spät nach einer Entlassung von stationären Patienten mit COVID-19 symptomatische VTE auftreten können. Die Autoren berichten über 2,6 % der Patienten mit einer symptomatischen VTE 42 Tage nach der Entlassung [11].

## Risikofaktoren

Die Risikofaktoren für konventionelle VTE sind gut bekannt, allerdings ist ihre Anwendung bei COVID-19-Patienten bisher nicht validiert worden. In Tab. 1 sind die wichtigsten intrinsischen und extrinsischen Risikofaktoren nach derzeitigem Wissenstand zusammengefasst [12]. Besonderes Interesse gebührt der Adipositas als Risikofaktor, weil mittlerweile bekannt ist, das Adipöse ein doppelt so hohes Risiko für eine schwere Erkrankung haben und ein um 50 % erhöhtes Todesrisiko [13] .

**Table 1:** 

Intrinsische Risikofaktoren	Extrinsische Risikofaktoren
Alter	Keine Thromboprophylaxe
Übergewicht	Therapie auf Intensivstation
Krebsanamnese	Ausgeprägte Lungenschäden
Frühere TVT oder PE	Hyperinflammation
Venöse Insuffizienz	Schwere Hypoxämie

*TVT* tiefe Venenthrombose, *PE* Pulmonalembolie

Das adipöse Gewebe kann als potentes inflammatorisches Reservoir für die Replikation von SARS-Cov‑2 dienen, weil die Adipozyten reichlich Angiotensin-Converting-Enzym-2-(ACE2)-Rezeptoren tragen. Außerdem besteht bei Adipösen eine Low-grade-Inflammation, die mit hohen Leptin-Spiegeln mit proinflammatorischen Wirkungen, niedrigen Adiponektin-Spiegeln mit antiinflammatorischen Effekten sowie mit einem prokoagulatorischen Status einhergeht.

Darüber hinaus finden sich bei adipösen Patienten höhere Interleukin-6- (IL-6) und Tumornekrosefaktor-alpha(TNF-α)-Spiegel im Blut und die Natural-Killer-Zellen (NK) werden zu nicht zytotoxischen NK-Zellen polarisiert. Viele Autoren empfehlen, dass adipöse und schwer adipöse Patienten als Hochrisikopatienten für eine COVID-19-Erkrankung eingestuft werden. Sowohl die Adipositas als auch COVID-19 scheinen einige gemeinsame metabolische und inflammatorische Reaktionswege zu teilen [14].

## Pathogenese

Die Pathogenese der sog. Koagulopathie bei der COVID-19-Erkrankung scheint auf einer Interaktion zwischen Thrombose und Inflammation zu beruhen, was zu einem hyperkoagulatorischen Status führt [15]. Hämostase und das Immunsystem ergänzen einander, um einen Schutz und Begrenzung der Ausbreitung von Pathogenen gewährleisten zu können. Die physiologische Immunothrombose kann sich zu einer Dysregulation steigern, indem es zu einer exzessiven Bildung von immunologisch mediierten Thrombi und zur Ausbreitung vor allem in der Mikrozirkulation kommt.

Die Immunothrombose scheint ein wichtiger pathologischer Mechanismus bei COVID-19 zu sein, bei dem die Aktivierung der Immunzellen, eine exzessive Koagulation und endotheliale Dysfunktion zum prothrombotischen Status beitragen [16] . Hauptmerkmal der Immunothrombose ist die Interaktion zwischen der Hämostase und dem angeborene Immunsystem, vor allem zwischen Monozyten, Makrophagen und Neutrophillen. Die vaskuläre Schädigung bei COVID-19 wird induziert durch die Endozytose von SARS-CoV‑2 in den Wirtszellen, was zu einer Pyroptose führt. Die Pyroptose ist eine extrem inflammatorische Form des programmierten Zelltods. Sie endet mit einer Zell-Lyse und der Freisetzung von diversen Damage-Associated Molecular Patterns (DAMPs) wie Adenosintriphosphat (ATP), Nukleinsäuren und Inflammasomen [17]. Die Pyroptose setzt nichtkapsulierte virale RNA und Proteine frei, die die umliegenden Zellen befallen und auf diese Weise das inflammatorische Milieu verstärken.

## Serologische Marker

Serumspiegel von D‑Dimer, Lymphozyten, Fibrinogen und Prothrombinzeit sind wichtige Laborparameter für die Schwere der Erkrankung und für eine VTE [6]. D‑Dimer-Spiegel sind bei Patienten mit VTE signifikant erhöht, die Lymphozytenzahl ist signifikant erniedrigt, Prothrombinzeit verlängert und Fibrinogen erhöht. Erhöhte D‑Dimer-Spiegel über 1,5 ng/ml (Referenzwerte bis 0,5 ng/ml) sind ein Zeichen für die Aktivierung der Koagulation und Fibrinolyse und ein guter Indikator für die Identifizierung von Hochrisiko-Populationen mit VTE.

Der D‑Dimer-Test kann als ein hochsensitiver Test für die Erkennung von aktiven thrombotischen Prozessen genutzt werden, allerdings mit einer niedrigen Spezifität [15]. IL‑6 ist erhöht bei Patienten mit schwerem COVID-19-Verlauf – als Marker für eine hyperinflammatorische Reaktion.

Viele Studien haben eine Assoziation zwischen erhöhten IL-6-Spiegeln und einem gesteigerten Risiko für vaskuläre Thrombosen und VTE gezeigt [18] . Die kombinierte Testung von D‑Dimer und IL‑6 bietet die höchste Sensitivität (bis 96,4 %) und Spezifität (bis 93,3 %) für eine mögliche frühe Voraussage der Schwere der COVID-19-Erkrankung[19].

## Ungleichgewicht der ACE2-Regulation

Eine Infektion mit SARS-CoV‑2 ist vermittelt durch das Binden des viralen Spike-Proteins an den ACE2-Rezeptor [20], der nicht nur in den Pneumozyten, sondern auch an am kardiovaskulären System breit exprimiert ist [21]. Das Eindringen des Virus in das alveoläre Epithelium via ACE2-Rezeptor führt zur Freisetzung von inflammatorischen Zytokinen wie IL‑6, TNF‑α ect. und Chemokinen, die ihrerseits Epithelzellen, Monozyten und Neutrophile aktivieren [22].

Auf der anderen Seite können Endothelzellen durch ihren ACE2-Rezeptor direkt vom Virus infiziert werden, was zu einer Endothelaktivierung und Dysfunktion führen kann. Sie äußert sich mit Einschalten der Koagulationskaskade, mit Bildung von Thrombin und Fibrin-Thrombi. Studien haben gezeigt, dass IL‑6 als Indikator der Inflammation bei vielen COVID-19-Patienten erhöht ist und eine klare Korrelation mit den Fibrinogenspiegeln zeigt, was wiederum die Theorie der inflammatorischen Thrombose unterstützt [23].

Durch die Stimulierung des ACE2-Rezeptors kommt es zu Störungen im Renin-Angiotensin-System und folglich zu Vasokonstriktion und Freisetzung inflammatorischer Zytokine [24]. Der prothrombotische Status bei COVID-19-Erkrankung kann mit der gesteigerten Koagulation, verminderten Fibrinolyse und immunologischen Effekten erklärt werden. Die Koagulation stellt eine komplexe biologische Kaskade dar und umfasst neben der Hämostase auch einen vaskulären Spasmus und Thrombozyten-Aktivierung. Eine reduzierte Fibrinolyse ist beschrieben bei Patienten mit COVID-19 [25]. Das fibrinolytische Plasminogen Activator Inhibitor 1 (PAI-1) ist bei COVID-19 erhöht, sowohl bei Intensiv- als auch bei Nicht-Intensivpatienten [26].

## Rolle der Thrombozyten

Die aktivierten Endothelzellen exprimieren eine Reihe von Proteinen wie P‑Selektin und Adhäsionsmoleküle, die ihrerseits eine Rekrutierung von Thrombozyten und Leukozyten zur Folge haben. Aktivierte Thrombozyten setzen eine Reihe von bioaktiven Molekülen frei (Adenosindiphosphat [ADP], Polyphosphate, Koagulationsfaktoren) und immunologische Mediatoren (Komplement-Faktoren), die eine weitere Aktivierung des Immunsystems durch positiven Feedback bewirken [27].

Bei COVID-19 ist VWF deutlich erhöht und kann eine Neigung zur Thrombose anzeigen

Marker für die Thrombozytenaktivierung, wie z. B. P‑Selektin, sind bei Patienten mit COVID-19 erhöht, und P‑Selektin kann durch Induktion der Tissue-Factor(TF)-Expression in Monozyten zu einem prokoagulatorischen Phänotyp führen [26]. Das Glykoprotein Von-Wilebrand-Faktor (VWF) wird von aktivierten Endothelzellen, Thrombozyten oder vom Subendothelium gebildet und vermittelt die Adhäsion und die Aggregation der Thrombozyten. Bei COVID-19 ist VWF deutlich erhöht und kann eine Neigung zur Thrombose anzeigen. Die Thrombozyten haben eine sehr wichtige Rolle im angeborenen Immunsystem, indem sie das Komplement aktivieren und dadurch eine Schlüsselstellung in der COVID-19-Immunothrombose spielen [28].

## Hypoxie

Bei moderaten bis schweren COVID-19-Verläufen kommt es zu Hypoxie und diese kann zur endothelialen Dysfunktion und Hyperkoagulabilität führen [29]. Es kommt zur Freisetzung von inflammatorischen Zytokinen (TNF‑α, IL-2) Förderung der Thrombose durch endotheliale Sekretion von PAI‑1 und Aktivierung von Makrophagen.

## Vasokonstriktion

Endotheliale Dysfunktion äußert sich auch durch Verlust des vaskulären Tonus, und wenn es zu Vasokonstriktion kommt, wird ein prothrombotischer Status getriggert. Die Hypoxie bei schwerer COVID-19-Erkrankung kann den Cyklo-Oxygenase(COX)-Weg in den Endothelzellen aktivieren und dadurch zu Konstriktion der glatten Muskelzellen in den Gefäßen führen [30]. Der vaskuläre Tonus wird aber auch durch einen Hypoxie-unabhängigen Mechanismus reguliert, nämlich durch das Renin-Angiotensin-Aldosteron-System. Die Herunterregulierung des ACE2-Rezeptors durch den Befall mit SARS-CoV‑2 führt zusätzlich zu Vasokonstriktion [31].

## Zytokine und Chemokine

Eine schwere COVID-19-Erkrankung ist charakterisiert durch eine gesteigerte Aktivierung des angeborenen Immunsystems und erhöhte Inflammation [32]. In der Folge kommt es zu einer verstärkten und unkontrollierten Freisetzung von Zytokinen, ein Phänomen, das Zytokinsturm genannt wird [33]. Bei Patienten mit COVID-19 sind viele Zytokine und Chemokine erhöht, darunter IL‑6, Interferon(IFN)-gamma und IL‑2 [34]. IL‑6 bewirkt eine Steigerung der Produktion und der Aktivität der Thrombozyten, erhöht die Expression von TF in Endothelzellen und Monozyten und kann eine endotheliale Dysfunktion auslösen. IFN‑γ hat ähnliche Effekte mit prothrombotischer Wirkung. IL‑2 vermindert die Fibrinolyse, indem PAI‑1 hochreguliert wird [34].

## Komplement

Die Aktivierung des Komplementsystems ist gut dokumentiert bei COVID-19, die mit der Bildung des terminalen Membrane Attack Complex (MAC) einhergeht [35]. MAC kann zu einer Aktivierung der Thrombozyten mit nachfolgender endothelialer Schädigung und Sekretion von VWF führen. Die individuellen Komponenten des Komplements wirken prothrombotisch, z. B. kann C5a die Freisetzung von TF und PAI‑1 stimulieren und Neutrophile aktivieren, die ihrerseits für die gesteigerte Freisetzung von IL‑6 und IL‑8 und die Bildung von Neutrophil Extracellular Traps (NETs) verantwortlich sind [36]. Somit führt die Aktivierung des Komplements zur Verstärkung des prothrombotischen Phänotyps bei COVID-19-Erkrankung.

## Neutrophil Extracellular Traps

**Figure 1:**
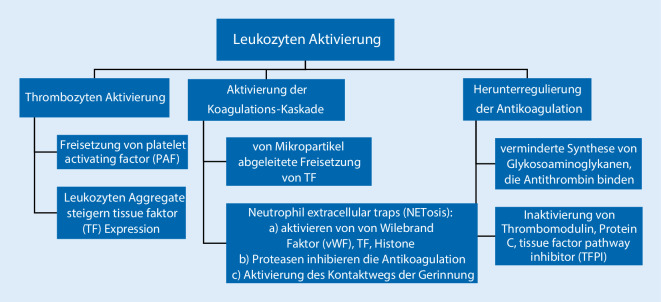

Neutrophile sind wichtige Mitspieler bei der Thrombusformation und migrieren schnell im Bereich der endothelialen Schädigung neben den Thrombozyten. Ein wichtiger Abwehrmechanismus ist die sog. NETosis, die von aktivierten Neutrophilen in Gang gesetzt wird mit dem Ziel, Pathogene zu beseitigen (Abb. 1). NETosis umfasst die extrazelluläre Freisetzung von NETs, die aus Chromatin und mikrobiellen Proteinen aufgebaut sind [37] .

NETs sind an der Pathobiologie der VTE maßgeblich beteiligt, und erhöhte Serumspiegel korrelieren mit der Mortalität [38]. NETs führen zu einer Steigerung der lokalen Inflammation und Ausdehnung der mikrovaskulären Thrombose. Erhöhte Spiegel von zirkulierenden Histonen und Myeloperoxydase-DNA bei schwerer COVID-19-Erkrankung wurden in mehreren Fallberichten gemessen [39]. Eine Studie mit Autopsie-gewonnenem Gewebe bei Patienten mit COVID-19 berichtete über NET-Aggregate in der Mikrovaskularisation, die in einer vaskulären Okklusion und Organschädigung resultierten [40]. Bildung von NETs kann die proinflammatorischen und prokoagulatorischen Faktoren bei COVID-19 verstärken und zu einem prothrombotischen Phänotyp beitragen.

## Zusätzliche thrombotische Mechanismen

Erhöhte Spiegel von Ferritin bei COVID-19 spiegeln eine Zellschädigung wider und können zur Inflammation beitragen. Hohe Ferritinspiegel können schädliche Effekte auf Mitochondrien haben und führen zur Freisetzung von reaktiven Sauerstoffradikalen, die den Zelltod auslösen [41]. Eine mitochondriale Dysfunktion der Thrombozyten kann zu Inflammation und prothrombotischem Status beitragen.

Erhöhte Antiphospholipid-Antikörper (APA) sind bei COVID-19 beschrieben, obwohl ihre Signifikanz nicht ganz klar ist [42]. APA können mit Endothelzellen, Leukozyten, Thrombozyten und Komplement interagieren, indem sie die Freisetzung von prothrombotischen Faktoren triggern [43].

Adipositas ist mit einem Zustand der langandauernden subakuten Inflammation verbunden und stellt einen Risikofaktor sowohl für COVID-19 als auch für VTE dar. Die Hypertrophie und die damit verbundene Dysfunktion der Adipozyten bewirkt eine Freisetzung von IL‑6, PAI‑1 und TF, die das Koagulationssystem aktivieren [44]. Mit der gesteigerten Produktion von Leptin und der verminderten Produktion von Adiponectin bei adipösen Menschen wird die Thrombozytenaggregation gefördert.

## Therapie und Prophylaxe der COVID-19-VTE

Für die Therapie der COVID-19-VTE werden eine Reihe von Substanzen eingesetzt – wie unfraktioniertes Heparin, niedermolekulare Heparine („low molecular weight heparin“, LMWH), direkte orale Antikoagulanzien (DOAK), Aggregationshemmer, Faktor-XII-Inhibitoren, thrombolytische Agenten, Nafamostat, Anti-Komplement und Anti-NET-Medikamente sowie IL-1-Rezeptor-Antagonist [45]. Zurzeit laufen mehrere randomisierte kontrollierte Studien (RCTs), die verschiedene Therapien, Kombinationen und Prophylaxe-Regime untersuchen. Interimistische Ergebnisse von Multiplatform-RCTs bezüglich VTE-Prophylaxe zeigen, dass bei moderater COVID-19-Erkrankung (hospitalisiert, nicht intensiv) therapeutische Dosen von LMWH besser als prophylaktische Dosen zu sein scheinen – mit positiven Effekten auf Morbidität und Mortalität und weniger als 2 % schweren Blutungen.

Bei schwerem COVID-19-Verlauf (Intensivpatienten) verbesserte sich der Verlauf durch therapeutische Dosen Heparin nicht, und sie scheinen im Vergleich zu den prophylaktischen Dosen unterlegen zu sein [46]. Die erste starke Real-world-Evidenz kommt aus den USA, aus einer Beobachtungskohortenstudie, die eine frühere prophylaktische Antikoagulation im Vergleich zu keiner Antikoagulation bei hospitalisierten COVID-19-Patienten (nicht intensiv) untersucht hat [47]. Die sofortige Behandlung mit prophylaktischem Heparin war mit einer 34 %igen Reduktion des relativen 30-tägigen Mortalitätsrisikos verbunden und mit einer absoluten Risikoreduktion von 4,4 %. Es fand sich kein gesteigertes Blutungsrisiko unter prophylaktischer Antikoagulation.

Für ambulante COVID-19-Patienten wird von den Leitlinien keine VTE-Prophylaxe empfohlen

Zurzeit empfehlen alle Leitlinien der medizinischen Gesellschaften eine VTE-Prophylaxe vorzugsweise mit LMWH für jeden stationären COVID-19-Patienten. Für ambulante COVID-19-Patienten wird von den Leitlinien keine VTE-Prophylaxe empfohlen. Für stationäre Patienten, die entlassen wurden, wird in manchen Leitlinien eine prophylaktische Antikoagulation für 1–2 Wochen empfohlen, wenn zusätzliche Risikofaktoren bestehen [15] . Sobald die Daten der RCTs vorliegen, werden sicherlich die Therapie- und Prophylaxe-Empfehlungen adaptiert und neu herausgegeben.

## Möglicher Mechanismus der Thrombozytopenie nach COVID-19 Impfung

Deutschen und norwegischen Wissenschaftlern ist es gelungen, Antikörper in geimpften Individuen nachzuweisen, die vermutlich die Thrombozyten angreifen und für die thrombotischen Ereignisse verantwortlich sein können [49]. Unabhängig davon stellte Merchand die Hypothese auf, dass die mRNA (Pfizer, Moderna) und Adenovirus (AstraZeneca, Johnson & Johnson) Vaccinen eine Synthese des COVID spike Proteins in Thrombozyten induzieren könnten und so eine Autoimmunreaktion gegen Plättchen ausgelöst werden könnte [50].)

## Fazit

COVID-19 ist eine systemische Erkrankung, gekennzeichnet durch Dysregulation des Immunsystems und einen hyperkoagulablen Status. Dieser Status ist Folge der direkten Virus-ausgelösten endothelialen Schädigung, Leukozyten- und Zytokinen-mediierte Aktivierung der Thrombozyten, Freisetzung von TF und NETosis, verstärkt durch eine Aktivierung des Komplementsystems. Die starke Aktivierung des Immunsystems durch die SARS-CoV-2-Infektion führt zu einer nicht regulierbaren Thrombose, die sich mit vielen Mikrothrombi in der Mikrovaskularisation, VTE und arteriellen Ereignissen präsentieren kann.
